# Dental Materia Medica

**Published:** 1866-04

**Authors:** Geo. Watt


### DENTAL REGISTER.

VOL. XX.] APRIL 1866. [NO. 4.

### Original Communications.

### DENTAL MATERIA MEDICA.

BY GEO. WATT.

IODINE, I = 126.

MOST of the iodine of commerce is obtained from the kelp of the west coast of Ireland, and the northern shores of Scotland. The kelp is obtained by burning sea-weeds, and is principally used by soap makers, on account of the carbonate of soda which it contains. The lye which remains, after the carbonate is removed by crystallization, contains considerable quantities of iodine, in the form of iodide of sodium.

PREPARATION.—The iodine lye, above described, is placed in a leaden still, and sulphuric acid is added to it. Sulphurous, hydro-sulphuric, and carbonic acids are evolved as gases; and sulphur is precipitated. It is then gently heated to 140°, and binoxyd of manganese is added. Iodine is evolved as a vapor, and is condensed in a series of spherical, double-mouthed, glass condensers. Those familiar with chemical notation, will understand the reactions of the process, by the following equation:

$$NaI + MnO_2 + 2SO_3 = NaO,SO_3 + MnO,SO^3 + I.$$

Properties.—Iodine is an elementary body, and, at ordinary temperatures, a crystalline solid. It is usually obtained in friable scales, of a grayish-black color, resembling micaceous iron ore. Though not a metal, it has a metallic lustre. It has an unpleasant acrid taste, and an odor something like chlorine, but less suffocating. Its melting point is 225°, and it volatilizes at 347°. Placed in boiling water, its vapor is given off with that of the water. Its vapor has a beautiful violet color, and is remarkable for its great specific gravity, being nearly nine times as heavy as atmospheric air. Iodine is readily soluble in alcohol and ether, and also in solutions of the iodides, but requires 7000 times its weight of water to dissolve it.

Characteristics.—Iodine may be generally recognized by the violet color of its vapor. This may be readily observed by dropping the iodine on a hot brick. It forms a blue compound with starch. Starch is a very delicate test for iodine; but the iodine must be free, and the mixture cold. When the iodine is combined, as with potassium or sodium, a little nitric acid should be added, to take the base, and the blue color will at once be developed by the addition of the starch solution.

Physiological Effects.—Iodine acts mainly, if not solely, by virtue of its chemical affinities. Locally, it is an irritant—almost an escharotic. It stains the cuticle a dark yellow, and causes redness and desquamation. Its affinities are nearly analogous to those of chlorine, but are less energetic. It is said to act as an *alterative*, a *liquefacient*, or *resolvent*; but these are only convenient terms by which to express our ignorance.

Modus Operandi.—Whether used externally or internally, iodine is readily absorbed. It may be found in the blood, sweat, urine, milk, saliva, and mucous secretions. Dr. Cogswell found it in the urine of a dog, five days after ceasing its administration. Cantu says it is never found free in the blood or secretions, but always as an iodide, or hydriodate, from which he infers that its action on the body is chemical. When absorbed in a free state, it is natural to infer, that there will be constitutional disturbance, as, by its affinity, it manifests its irritant action till it is neutralized by combination, that is, till it becomes an iodide. Hence, when its constitutional action, and not its local, is desired, it is more in accordance with chemical philosophy to use an iodide rather than free iodine. When taken into the circulation, iodine appears to produce, as Müller observes, "such an alteration in the composition of the tissues, that the affinities already existing are annulled, and new ones induced, so as to enable the vital principle—the power which determines the constant reproduction of all parts, in conformity with the original type of the individual—to effect the further restoration and cure."

USES.—Iodine may be useful to the dentist in the local treatment of chronic inflammation, or induration of the salivary glands, in dental periostitis, in alveolar abscess, in some morbid states of the antrum, in thickening of the mucous membrane, in tumors of the mouth, and in absorption of the gums and alveolar processes. The officinal tincture will answer very well for periostitis, thickening of membrane, and sometimes for abscess. For chronic catarrh, or inflammation of the lining membrane of the antrum, the compound tincture, very much diluted, is a good injection. For destroying the sac in alveolar abscess, a solution of iodine in creosote is a sovereign agent; and when not too concentrated, the same is an admirable application to the margins of the gums and alveolar processes, after the removal of all irritating and dead substances; but care must be taken that it is not applied too frequently. It is an escharotic; and, as a general rule, it is well to let the slough separate before a second application; and, frequently, milder preparations of iodine should follow the first application. To use externally, I prefer a colorless tincture of iodine, prepared by combining equal quantities of compound tincture of iodine and pure aqua ammonia. As combination takes place, the mixture becomes transparent, and will not then color the skin.

I do not think of any condition which will require the dentist to prescribe *iodine* internally. When its constitutional action is indicated, as in scrofulous or syphilitic diseases of the mouth, he will derive more benefit from the use of iodide of potassium.

### Iodide of Potassium, KI=165.

This salt, as its name imports, is composed of one equivalent of iodine and one of potassium. The equivalent of the former is 126, that of the latter, 39. Hence the salt contains 76.4 per cent of iodine, and 23.6 per cent of potassium.

Preparation.—The dentist will find it more convenient to buy than to prepare the salt. Iodine, iron, carbonate of potassa, and water, are used in its preparation. For the relative quantities and manipulations, the reader is referred to the United States Dispensatory. The iodine combines with the iron, and the iodide of iron, thus formed, decomposes the carbonate of potash, forming the carbonate of iron and the salt under consideration. The reactions are represented by the following equation:

$$FeI+KO,CO_2=FeO,CO_2+KI.$$

Properties.—Iodide of potassium is a white, shining, semi-transparent, inodorous salt, having an acrid taste. It melts at a red heat, and is vaporized at high temperatures, without decomposition. It is readily soluble in alcohol and water, requiring for its solution, but two thirds of its weight of water, at 60°. It contains no water of crystallization.

Impurities.—A reliable article can always be obtained from responsible apothecaries; but it is often adulterated. It is more likely to contain carbonate of potash than any other salt. This may be detected by its action on lime water. The pure salt produces no visible change; but if the carbonate is present, the lime water is rendered turbid, or milky, by the formation of carbonate of lime. Again, the iodide is soluble in alcohol, and the carbonate is not.

Physiological Effects, and Modus Operandi.—Locally, this salt is an irritant, but is not nearly so energetic in its action as free iodine. On this account it may be given, internally, in larger doses, and for a longer period, than iodine. Indeed, iodine can be introduced into the system much faster by the use of the iodide, than when given uncombined. A solution of albumen, fibrin, or gelatin, is not obviously changed by the addition of this salt; and as these are the most abundant organic constituents of the body, we may infer that the chemical action of the iodide, on the living tissues, is but slight.

To obtain a clear view of the action of this salt, as a remedial agent, it is necessary to bear in mind its peculiar properties. It is very soluble, and is, therefore, readily absorbed. It passes rapidly into the circulation. and may be detected in all the tissues and secretions. It is composed of two elements, both of which are characterized by strong affinities, some of them stronger than that by which they are held together. If the salt is decomposed, the potassium takes oxygen, and becomes potash, which is a general solvent of animal tissues. At the same time, the iodine is set free, and is thus able to exert its affinities. And as all chemical agents are peculiarly active in the nascent condition, the iodine and potash are both more energetic, than if carried, in their free state, to the point of action. Each one, as it were, holds the other quiet till the proper point is reached, and then lets it go to accomplish its work. Each element, by neutralizing the other, prevents its local, *irritant* action; and each is liberated, atom by atom, in obedience to stronger affinities, each particle being promptly saturated by the gratification of the affinity which liberates it. And this explains how it is, that such large doses of this salt can be taken for a long time, without local or constitutional disturbance.

The affinities of iodine and potassa, are sufficient to account for all the phenomena observed in the remedial action of the salt. Highly soluble compounds are the natural results; and these are as naturally carried out by the various excretories. Let us suppose that the iodide is administered for the arrest or removal of morbid growths, or to relieve tertiary syphilis. The latter is often spoken of as a disease of the bones. But does that expression convey the whole truth? Is there not a disease of the formative fluids, from which bony tissue is deposited? Now, if these morbid particles are arrested by the affinities of the elements of the salt, held in solution, and carried out through the various secretions, it is evident they will not further build up the morbid development. And as the particles of morbid structures, like those of normal tissues, perform their functions and pass away, unless new ones are furnished in their places, the abnormal solid is carried off, little by little. The diseased growth is literally starved to death, and carried out by the scavengers of the system. It is this action of the remedy, which has induced some writers to call it a *resolvent*, or *liquefacient*.

In some of the above remarks, I have sacrificed technicality, to a desire to be understood by beginners, and those whose opportunities have not been such as they desired. They are not written for the critic, though, of course, he may use his pleasure in regard to them.

Uses.—Whenever the dentist has to treat a diseased condition, that calls for the constitutional action of iodine, he will do better to use iodide of potassium. He will need it in treating chronic, non malignant diseases of the antrum. It seems to exert a special influence on the nasal mucous membrane. Pereira has observed that the pocket-handkerchiefs of those using this salt, "acquire a distinct odor of iodine."

In absorption of the alveolar processes, especially when accompanied with subacute, or chronic periostitis, its use is indicated. And in all cases, when the mouth gives evidence of syphilitic or scrofulous disease, it is well to administer it. As a local application, it is not so useful as free iodine.

Administration.—It should be given in solution, and usually immediately after eating. It may be taken in sweetened water, or almost any way the patient may fancy. The average dose is from 4 to 10 grains. Many use much larger doses, but I have not found it profitable to do so. For an adult, I frequently prescribe a solution of a dram of the salt to an ounce of water, and direct the patient to take a teaspoonful three times a day.

